# Original XPC^TM^ Effect on *Salmonella* Typhimurium and Cecal Microbiota from Three Different Ages of Broiler Chickens When Incubated in an Anaerobic *In Vitro* Culture System

**DOI:** 10.3389/fmicb.2017.01070

**Published:** 2017-06-13

**Authors:** Si Hong Park, Sun Ae Kim, Sang In Lee, Peter M. Rubinelli, Stephanie M. Roto, Hilary O. Pavlidis, Donald R. McIntyre, Steven C. Ricke

**Affiliations:** ^1^Center for Food Safety and Department of Food Science, University of Arkansas, FayettevilleAR, United States; ^2^Diamond V, Cedar RapidsIA, United States

**Keywords:** poultry, *Salmonella*, *in vitro*, microbiome, sequence

## Abstract

Feed supplements are utilized in the poultry industry as a means for improving growth performance and reducing pathogens. The aim of the present study was to evaluate the effects of Diamond V Original XPC^TM^ (XPC, a fermented product generated from yeast cultures) on *Salmonella* Typhimurium ST 97 along with its potential for modulation of the cecal microbiota by using an anaerobic *in vitro* mixed culture assay. Cecal slurries obtained from three broiler chickens at each of three sampling ages (14, 28, and 42 days) were generated and exposed to a 24 h pre-incubation period with the various treatments: XPC (1% XPC, ceca, and feeds), CO (ceca only), and NC (negative control) group consisting of ceca and feeds. The XPC, CO, and NC were each challenged with *S.* Typhimurium and subsequently plated on selective media at 0, 24, and 48 h. Plating results indicated that the XPC treatment significantly reduced the survival of *S.* Typhimurium at the 24 h plating time point for both the 28 and 42 days bird sampling ages, while *S.* Typhimurium reduction in the NC appeared to eventually reach the same population survival level at the 48 h plating time point. For microbiome analysis, Trial 1 revealed that XPC, CO, and NC groups exhibited a similar pattern of taxa summary. However, more Bacteroidetes were observed in the CO group at 24 and 48 h. There were no significant differences (*P* > 0.05) in alpha diversity among samples based on day, hour and treatment. For beta diversity analysis, a pattern shift was observed when samples clustered according to sampling hour. In Trial 2, both XPC and NC groups exhibited the highest Firmicutes level at 0 h but the Bacteroidetes group became dominant at 6 h. Complexity of alpha diversity was increased in the initial contents from older birds and became less complex after 6 h of incubation. Beta diversity analysis was clustered as a function of treatment NC and XPC groups and by individual hours including 6, 12, 24, and 48 h. Overall, addition of XPC influenced microbiome diversity in a similar fashion to the profile of the NC group.

## Introduction

Prebiotics are often used in the poultry industry as a replacement for antibiotic growth promoters (AGPs); they are expected to maximize growth, while minimizing pathogen invasion by selectively stimulating beneficial bacteria (Roberfroid, 2007). Prebiotics are defined as substances that travel past the upper gastrointestinal tract (GIT; resisting hydrolysis by gastric enzymes and degradation by acidic pH) remaining intact, while acting as selective substrates for beneficial bacteria in the lower GIT and subsequently improving host health (Gibson and Roberfroid, 1995; Roberfroid, 2007). However, the definition of a prebiotic and similar acting compounds is being re-evaluated as more has become understood about the gastrointestinal microbiome and its corresponding response to these types of compounds (Hutkins et al., 2015; Ricke, 2016; Ricke et al., 2017). With this in mind, there are several feed supplements available that do not fit the more stringent version of the prebiotic definition as set by Gibson and Roberfroid (1995), yet still do appear to provide advantageous benefits to host health. These feed supplements are known as prebiotic-like compounds (Roto et al., 2015). A common prebiotic-like compound is a *Saccharomyces cerevisiae* fermentation product (SCFP), which contains the fermentation products of *S. cerevisiae* along with metabolites plus the media used in the fermentation (Original XPC^TM^ Diamond V, Cedar Rapids, IA, United States). Research regarding XPC has been conducted in several different animal model systems, both *in vivo* and *in vitro*, to investigate its effects on the health of host (Gao et al., 2008; Osweiler et al., 2010; Price et al., 2010; Rubinelli et al., 2016).

Chickens and other poultry products are some of the more popular meat products throughout the world but live birds can become colonized by pathogenic bacteria during the growth cycle of the birds. *Salmonella* is one of the primary pathogens causing foodborne disease associated with poultry products consumed by humans (Hedican et al., 2009; Foley et al., 2011, 2013; Finstad et al., 2012). *Salmonella* in chickens was identified as the food-etiologic agent responsible for a substantial percentage of the foodborne outbreaks in the United States in 2013 (Centers for Disease Control and Prevention (CDC), 2015). Therefore, *Salmonella* contamination continues to be a serious problem in the poultry industry and research on effective control measures during live bird production remains a critical component for implementation of any overall intervention strategy for poultry production.

The current research utilizes an anaerobic *in vitro* mixed cecal culture assay to simulate the chicken hindgut to quantify *Salmonella* survival in the presence of cecal microbiota. This *in vitro* methodology allows for a more direct assessment on the cecal performance of XPC, while reducing confounding host variables (for example, host immune response) and being more cost efficient (Polli, 2008). A previously conducted *in vitro* study by Rubinelli et al. (2016) revealed that *Salmonella* inhibition occurred in conjunction with short chain fatty acid (SCFAs) production by cecal microbiota during incubation. However, they only examined one time point, inoculated with cecal contents from mature birds, and did not characterize the microbiome responses. A temporal effect on the cecal microbiota as the bird matures has been reported in previous studies (Scupham, 2009; Danzeisen et al., 2013; Oakley et al., 2014). To take this into account, the current study retrieved cecal inocula from birds at three different ages to evaluate the potential of XPC on the inhibition of *Salmonella* in the presence of poultry host ceca at different stages of microbiome development. In addition, the cecal microbial populations and species diversity within the cecal microbiota as a result of XPC addition and host maturity were also assessed.

## Materials and Methods

### Experimental Design

This experiment consisted of two independent trials, each with three biological replicates (individual birds) utilized at each of the three cecal sample collection time points: 14, 28, and 42 days.

### *Salmonella* Typhimurium Preparation

This study used a chicken isolate of *S.* Typhimurium (strain ST 97, Dr. Billy Hargis, Poultry Health Laboratory, University of Arkansas) resistant to nalidixic acid (NA) to selectively distinguish this specific strain from a mixed microbial background. *Salmonella* Typhimurium cultures were grown in 6 mL Luria Bertani (LB) broth supplemented with 20 μg/mL NA for 16 h with shaking at 37°C and washed in phosphate-buffered saline (PBS) three times and resuspended in 1 mL PBS. Optical density was measured at 600 nm with a spectrophotometer (Beckman Coulter, Inc., Brea, CA, United States).

### Broiler Chicken and Cecal Preparation

Animal handling and procedures were conducted in accordance with guidelines of the University of Arkansas’s Institutional Animal Care and Use Committee (IACUC). Ten male broiler chicks (per trial) were obtained from Cobb-Vantress, Inc. (Siloam Springs, AR, United States), grown in a pre-disinfected Horsfall unit, and provided antibiotic-free corn-based poultry feed and water *ad libitum.* Broilers were randomly tagged with leg bands, euthanized by CO_2_ asphyxiation, and their ceca were collected aseptically into sterile sample bags (VWR, Radnor, PA, United States). The ceca were transferred into an anaerobic chamber (Coy Laboratory Products, Grass Lake, MI, United States), the cecal contents (0.1 g) were weighed, and subsequently diluted to a 1:3000 concentration in anaerobic dilution solution [ADS, 0.45 g/L KH_2_PO_4_, 0.45 g/L (NH_4_)_2_SO_4_, 0.9 g/L NaCl, 0.1875 g/L MgSO_4_-7H_2_O, 0.12 g/L CaCl_2_-2H_2_O, 0.1% resazurin, 0.05% cysteine-HCl, and 0.4% CO_2_-saturated sodium carbonate]. The ADS was prepared as Bryant and Robinson (1961) originally described, with cysteine-HCl added prior to autoclaving as described in Shermer et al. (1998).

### *In Vitro* Incubation

The *in vitro* cecal incubation procedure was carried out as described previously (Donalson et al., 2007; Rubinelli et al., 2016). Autoclaved serum bottles (100 mL) were prepared containing 0.5 g Torres Chick Starter (**Table 1**) and 1% XPC (yeast cell wall fermented product). A 40 mL volume of diluted cecal contents was added to each serum bottle. All serum bottles were placed directly into incubation at 37°C for a 24 h pre-incubation and inoculated with *S.* Typhimurium at a final concentration of 10^7^ CFU/mL after the pre-incubation. Contents were subsequently plated on LB+NA+novobiocin (NO) media to serve as the baseline (0 h incubation). Repeated plating occurred at 24 and 48 h post-inoculation to determine *S.* Typhimurium survival. Each treatment group containing cecal contents, feed, and XPC was compared to three control treatments: (1) negative control (NC, cecal, and feed), (2) cecal only control (CO), and (3) XPC, cecal, and feed. Aliquots of samples (2 mL) were collected at 0, 6, 12, 24, and 48 h for microbiome analysis of the corresponding NC, CO, and XPC treatments.

**Table 1:** Ingredient composition of the Torres Chick starter diet.

Ingredient	Composition of total (%)
Corn	63.07
Soybean meal	25.75
Fat	2.85
Calcium carbonate	1.03
Dicalcium phosphate	1.10
Salt	0.40
DL methionine	0.28
Trace minerals	0.10
Choline chloride	0.22
Vitamin premix	0.20
ProPack	5.00

### Microbiome Analysis via Illumina MiSeq

Extraction of cecal DNA from aliquots of samples for microbiome analysis was conducted via QIAamp Fast DNA Stool Mini Kit according to the manufacturer’s protocol (Qiagen, Valencia, CA, United States). The final step of the DNA extraction deviated from the manufacturer’s protocol with DNase/RNase-Free distilled water being used as a substitute in place of the elution buffer that had been provided. Concentrations and purity of the cecal DNA samples were measured using a Nanodrop ND-1000 (Thermo Scientific, Waltham, MA, United States).

A polymerase chain reaction (PCR) was used to amplify the V4 region of the 16S rRNA gene with dual-indexed primers via an Eppendorf Mastercycler pro S (Eppendorf, Hamburg, Germany) according to the methodology described by Kozich et al. (2013). Confirmation of PCR amplicons was conducted on 1% agarose gel. Invitrogen SequalPrep kit (Life Technologies, Carlsbad, CA, United States) was utilized for the normalization of PCR amplicons according to the manufacturer’s protocol. The samples from each of the wells were pooled together. For quantification of the pooled samples, the Eppendorf realplex Mastercycler ep gradient S (Eppendorf) was utilized via the KAPA Library Quantification Kit (KAPA Biosystems, Wilmington, MA, United States) according to the manufacturer’s protocol (*R*^2^ = 0.999; efficiency = 96%). The length of the amplicon fragments was evaluated using the Agilent Bioanalyzer. Amplicon lengths were diluted to 4 nM, combined with prepared PhiX Control, and subsequently loaded into an Illumina MiSeq reagent cartridge.

### Sequence and Statistical Analysis

Sequencing (FASTA format) files were downloaded from the Illumina Basespace website. Sequence analysis, classification of operational taxonomic units (OTUs), and species diversity and richness (Chao1 and Shannon diversity index) were calculated via the quantitative insights into microbial ecology (QIIME 1.9.0; Caporaso et al., 2010) pipeline. Sequencing quality filtering over 99% were utilized for downstream analysis and chimera sequences were removed based on the ChimeraSlayer (identify_chimeric_seqs.py) using a BLAST. Taxonomic levels were identified based on the Greengenes database (gg13_5) with 97% identity. UniFrac principal coordinates analysis (PCoA) plots, generated via QIIME, were used to determine the multidimensional distances reflecting similarities and differences among samples based on age and treatment. Sequences with less than 10,000 reads were excluded from analysis.

The JMP^®^ Pro 12 (SAS Institute, Cary, NC, United States) software was utilized for statistical analysis. One-way analysis of variance (ANOVA) and Student’s *t*-tests evaluated the statistical significance among microbial relative abundance with a significance level of *P* < 0.05. Repeated measures analysis using the Fit Model platform in JMP were also performed to take into account the fact that the same *in vitro* cultures were sampled at multiple time points. A split-plot design was used. These model fitting results are discussed in each of the following subsections.

## Results and Discussion

### *Salmonella* Typhimurium Survival in Treatment with XPC

The first objective of the current research was to examine the impact of XPC on *S.* Typhimurium in a mixed *in vitro* cecal culture assay. Poultry ceca contain the largest number of bacteria due to the relatively slow digesta transit time (Salanitro et al., 1974). As the bird matures, the composition of the cecal bacteria become more diversified and reach concentrations that allow them to maximize their metabolic fermentative activities in an anaerobic environment (Roto et al., 2015). The *in vitro* assay in the current study based on our previous studies (Donalson et al., 2007; Rubinelli et al., 2016) attempts to simulate the environment of the chicken ceca, providing chicken feed as the nutrient supply for the cecal contents while maintaining the anaerobic environment. The majority of bacteria in the poultry ceca are strictly anaerobic, and have traditionally been enumerated using anaerobic jars and selective media (Fan et al., 1995; Ricke and Pillai, 1999).

The methods in the current study demonstrate the potentially synergistic effects between XPC and the cecal bacterial populations based on the assumption that XPC is maintaining its activity until it reaches the ceca. Fermentation metabolites of XPC includes yeast cell wall fragments such as beta-glucans and mannan-oligosaccharides, yeast cell residues, and post-fermented growth medium residues (Shen et al., 2011). Based on previous studies, the assay utilized a 24 h adaptation period for each sample containing cecal contents and poultry feed (Donalson et al., 2007; Rubinelli et al., 2016). This adaptation period allowed the cecal bacteria to ferment and continue the metabolism of substrates from both poultry feed alone, and in combination with XPC supplementation, prior to being challenged with *S.* Typhimurium (Rubinelli et al., 2016). The 24 h adaptation period is necessary to detect the effectiveness of XPC based on its potential mechanistic activity being associated with the cecal microbiota in some manner (Rubinelli et al., 2016).

**FIGURE 1:**
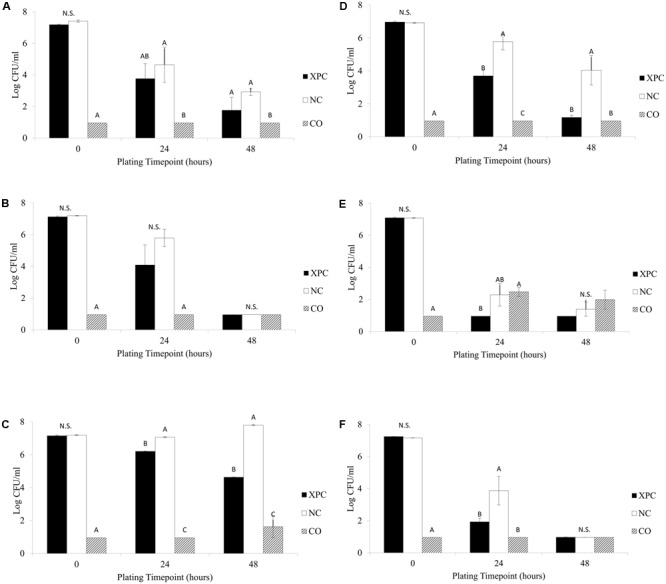
*Salmonella* Typhimurium survival among the treatments (XPC, XPC treatment; NC, negative control; CO, cecal only). **(A)** Trial 1–14 days old chickens, **(B)** Trial 2–14 days old chickens, **(C)** Trial 1–28 days old chickens, **(D)** Trial 2–28 days old chickens, **(E)** Trial 1–42 days old chickens, and **(F)** Trial 2–42 days old chickens. Differing letters indicate significant differences (*P* < 0.05).

Previous research has indicated that XPC can inhibit various pathogens as well as increase the levels of antibodies in blood samples (Gao et al., 2008; Jensen et al., 2008; Feye et al., 2016). In addition, Rubinelli et al. (2016) suggested that inclusion of XPC to diets may influence cecal microbiota *in vitro* fermentation and may exhibit a *Salmonella* inhibition effect in broilers and layers. The current study confirmed this pattern, resulting in a 0.5 to 3.0 log *S.* Typhimurium reductions exposed to the XPC treatment compared to the levels recovered from NC treatment. At the 14 days sampling age, there were no significant differences in the *S.* Typhimurium survival observed in the XPC treatment when compared to the NC group in either trial, although there were numerical (approximately 1.0 log, *P* > 0.05) reductions (**Figures 1A,B**). However, repeated measures analysis indicated that time of incubation and treatment had a significant effect (*P* < 0.05) on *Salmonella* survival at 14 days sampling age. The results obtained at the 28 days sampling age varied among trials. All reductions observed in the XPC treatment were significant (*P* < 0.05) at the 24 and 48 h plating time points at the 28 days sampling age (**Figures 1C,D**). The reductions observed in the XPC treatment in Trial 2 were approximately 2.0 and 3.5 logs greater at the 24 and 48 h plating time points, respectively, as compared to the Trial 1. In Trial 1, the XPC treatments resulted in approximately 1.0 and 3.0 log reductions in recovered *S.* Typhimurium at both the 24 and 48 h time points as compared to the NC. In Trial 2, exposure to the XPC treatment resulted in *S.* Typhimurium population levels 2.0 and 3.0 logs lower than the NC treatment in the 24 and 48 h plating time point respectively, with the recovery below the limit of detection (LOD) of 10 CFU/ml at the 48 h plating time point (**Figures 1B,F**). The variation observed between the two trials at the 28 days sampling age is potentially an indicator to the degree of microbial development (and, in turn, inconsistency) in the cecal microbiome composition among birds. Repeated measures analysis indicated a time effect on *Salmonella* reduction in Trial 2 (*P* < 0.05), but not in Trial 1, while treatment effect exhibited a significant difference in both trials.

At the 24 h plating time point samples from the 42 days birds (**Figures 1E,F**) there were numerical *S.* Typhimurium reductions (between 1.0 and 2.0 logs) occurring in both trials when comparing the XPC treatment and NC, with Trial 2 incubations exhibiting significant differences (*P* < 0.05, **Figure 1F**). At the 42 days sampling age, there were no significant differences (*P* > 0.05) observed among any treatments for the 48 h plating time point, as the XPC treatment responses were below the LOD in both trials. What is interesting is that although there were numerical differences between the XPC and NC treatments, presence of cecal contents was able to reduce the level of *S.* Typhimurium present to the LOD by the 48 h plating time point. This suggests the potential of the cecal microbiome adapting to the environment and being able to possibly out compete *S.* Typhimurium for nutrients and/or generate an unfavorable environment by the production of SCFA (Fooks and Gibson, 2002; Rubinelli et al., 2016). On the *Salmonella* reductions, repeated measures analysis indicated that there was no significant differences (*P* > 0.05) based on the treatments but incubation time was a statistically significant factor in Trial 1 (*P* < 0.05, **Figure 1E**). The same analysis indicated both treatment and time caused considerable *Salmonella* reductions in Trial 2 (*P* < 0.05) (**Figure 1F**).

**FIGURE 2:**
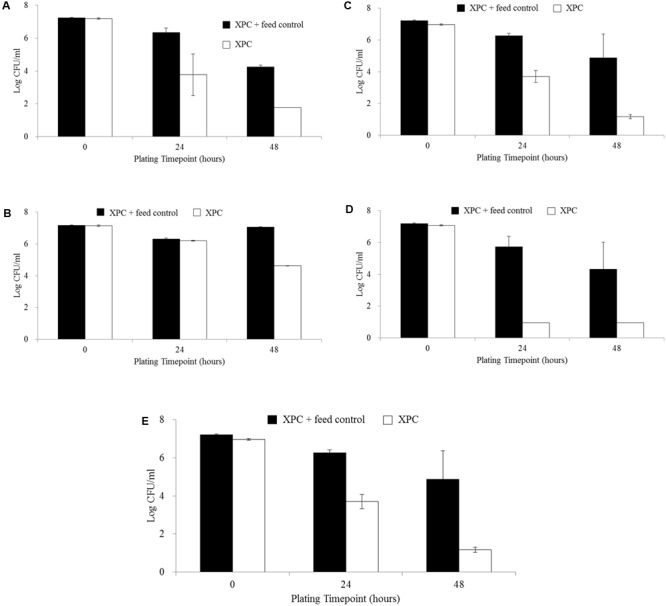
*Salmonella* Typhimurium survival comparing XPC + Feed Control and XPC treatment: **(A)** Trial 2–14 days chickens, **(B)** Trial 1–28 days chickens,**(C)** Trial 2–28 days chickens, **(D)** Trial 1–42 days chickens, and **(E)** Trial 2–42 days chickens. No figure for Trial 1–14 days chickens is presented as data for XPC + Feed Control was not collected at this time point.

The XPC + Feed control (containing no cecal slurry) resulted in higher levels of *S.* Typhimurium recovered compared to all treatments (XPC, NC, and CO) at all time points (14, 28, and 42 days; data not shown). The comparison of the results of the XPC treatment to the control containing only XPC + Feed control suggests the necessity of the cecal contents to exercise the mechanistic activity of XPC. The XPC + Feed control (containing no cecal slurry) revealed a 3.0 log reduction in the abundance of *S.* Typhimurium from the 0 h plating time point to the 48 h plating time points across all ages, while the XPC treatments (containing cecal slurry, feed, and XPC) in both trials exhibited much greater total log reductions (4.0 to 6.0 logs, *P* < 0.05; **Figures 2A–E**). Repeated measures analysis indicated that all trials and time points (**Figures 2A–D**) had significant differences (*P* < 0.05) based on both time and treatment (XPC) effects, except for the 42 days time point of Trial 2 (**Figure 2E**), which exhibited a time effect but no significant difference (*P* > 0.05) for treatment effect.

### Cecal Microbiome Analysis

Other studies involving various animal models indicate that XPC supplementation dose elicits an influential “host” factor that results in detectable changes in GIT morphology, immunologic response, growth performance, and pathogen reduction (Gao et al., 2008, 2009; Jensen et al., 2008; Osweiler et al., 2010). What has been recognized in the current research along with previous work is that feed supplements, aside from vitamins (Luo et al., 2013), thus far appear to have little general impact on the composition of the cecal microbiome (Danzeisen et al., 2013; Oakley et al., 2014).

There were significant observations regarding the successional changes in microbiome complexity that have not been observed with other feed supplements (different soy percentage and organic acids) (Lu et al., 2003; Danzeisen et al., 2013; Oakley et al., 2014). With the shift in diversity that was detected, there may also be a related shift in the physiological functions performed by the microorganisms present (Lu et al., 2003).

In poultry, the most vulnerable time in the maturation to market age is early on during the life of the bird when the intestinal tract is continuing to change both anatomically and physiologically, as well as the establishment of various bacterial groups along the intestinal epithelium (Schleifer, 1985; Iji et al., 2001; Brisbin et al., 2008). The stability of the cecal microbiota is directly related to age, as suggested by the increased variability observed among cecal microbiota composition in younger chickens (14 days) when compared to more mature chickens (28 days; Torok et al., 2009). The transient bacterial populations in the cecal microbiota of younger animals indicate immaturity and potentially increased susceptibility to invasion by pathogenic bacteria while the stable diversity among cecal populations in a mature broiler GIT allows for increased protection from pathogen invasion (Lozupone et al., 2012). Culture-independent analytical methods to characterize a given environment has become commonplace as it allows the study of the microorganisms within that environment without prior culturing, thereby eliminating any biases introduced by culture-based methods (Langendijk et al., 1995; Ricke and Pillai, 1999; Amit-Romach et al., 2004; Wooley and Ye, 2010). Sequencing of samples can be used to assess the variation of both the microbial species diversity as well as the structure of the communities over time and space (Hamady et al., 2010). However, several factors can potentially influence the final results and must always be considered during analyses and interpretation. For example, since we did not include a bead beating step during the DNA extraction procedure, this could lead to a potential bias in the final composition of Gram-negative and -positive bacteria recovered in the sample to be sequenced (Knudsen et al., 2016). Likewise, the analysis of the 16S rRNA gene sequences used primers targeting only the V4 hypervariable region should provide the required unique properties for taxonomic distinction but still be considered sufficiently conserved with a domain region of differing evolutionary rates to make it an optimal phylogenetic marker (Case et al., 2007; Caporaso et al., 2011).

After filtering the sequences based on read quality and sample size, there were a total of 135 samples in each of the two trials (45 samples per treatment total: three biological replicates at three sampling ages, each with five microbiome sampling time points) of the V4 region of the 16S rRNA gene analyzed. Analytical information regarding the sequences generated revealed 25,013,102 and 20,856,668 total reads and error rates of 2.05 and 2.15% in Trials 1 and 2, respectively.

Shannon diversity index analysis based on alpha diversity revealed that the addition of XPC increased the diversity significantly (*P* < 0.05) as the birds became older in both trials (**Table 2**). Repeated measures analysis also indicated significant effects of both treatment and time (*P* < 0.05). When the Shannon diversity of each of the incubation time points were compared, XPC appeared to retain the alpha diversity in a stable manner throughout the incubation time in Trial 1 (**Table 3A**). However, in Trial 2, XPC decreased the diversity for the 24 h of incubation time, but increased diversity again by 48 h (**Table 3B**). Repeated measures analysis for Shannon diversity indicated significant effects for treatment (*P* < 0.05), but not for the time points of the cultures.

**Table 2:** Shannon diversity index based on treatment and sampling age within their respective trials.

	Treatment	Sampling age (d)		
		14	28	42
Trial 1	XPC	1.89^B^	2.31^AB^	2.35^A^
	NC	1.96	2.08	2.17
	CO	2.15	2.31	2.27
Trial 2	XPC	1.75^B^	1.91^AB^	2.04^A^
	NC	1.92	1.82	1.92
	CO	2.17	2.21	2.28

*Samples are analyzed for significant differences within a treatment at the various sampling ages.*

*XPC, XPC treatment; NC, negative control; CO, cecal only control.*

*Differing letters within a treatment reveal significant differences (*P* < 0.05).*

*Lack of letters indicates no significance difference among Shannon diversity indices within the treatment group.*

*Incubation medium included 0.1 g of cecal contents.*

**Table 3:** Shannon diversity index based on treatment and incubation time within their respective trials.

(A) Trial 1					
	**Day 14**				**Day 28**					**Day 42**				
	**0 h**	**6 h**	**24 h**	**48 h**	**0 h**	**6 h**	**12 h**	**24 h**	**48 h**	**0 h**	**6 h**	**12 h**	**24 h**	**48 h**

XPC	4.52	4.22	4.41	4.94	5.24	5.33	5.21	5.26	5.13	5.52	4.78	4.92	5.26	5.28
NC	4.73^a^	3.36^b^	4.33^ab^	3.85^b^	5.33^a^	5.48^a^	5.28^a^	4.37^b^	3.93^b^	5.51^ab^	5.74^a^	4.97^bc^	4.67^cd^	4.12^d^
CO	5.05^c^	5.24^bc^	6.04^a^	5.92^ab^	5.59	5.3	5.81	5.42	5.24	5.14^a^	5.45^a^	5.54^a^	4.36^b^	5.21^a^

**(B)** Trial 2					

	**Day 14**					**Day 28**					**Day 42**				
	**0 h**	**6 h**	**12 h**	**24 h**	**48 h**	**0 h**	**6 h**	**12 h**	**24 h**	**48 h**	**0 h**	**6 h**	**12 h**	**24 h**	**48 h**

XPC	5.25^a^	3.83^b^	1.96^c^	3.35^b^	3.28^b^	5.01^a^	2.12^c^	2.01^c^	3.81^b^	3.67^b^	5.01^a^	2.69^d^	3.16^c^	3.82^b^	3.70^b^
NC	–	3.90^a^	2.47^bc^	2.03^c^	2.96^b^	4.77^a^	3.60^ab^	2.32^c^	2.15^c^	2.76^bc^	5.20^a^	3.54^b^	2.44^cb^	2.16^d^	3.18^bc^
CO	4.67^a^	4.38^ab^	–	4.15^b^	4.24^b^	4.61	4.64	4.48	4.70	4.72	5.01	4.89	4.89	4.92	5.17

*Samples are analyzed for significant differences within a treatment at the various incubation time.*

*XPC, XPC treatment; NC, negative control; CO, cecal only control.*

*Differing letters within a treatment reveal significant differences (*P* < 0.05).*

*Lack of numbers indicates no significance difference among Shannon diversity indices within the treatment group.*

*Incubation medium included 0.1 g of cecal contents.*

In Trial 1, a significant time effect (*P* < 0.05) was indicated by repeated measures analysis for Firmicutes and Cyanobacteria in the NC treatment; Cyanobacteria and Actinobacteria in the CO treatment; and Firmicutes, Proteobactera, and Bacteroidetes in the XPC treatment. In Trial 2, repeated measures analysis indicated a significant time effect (*P* < 0.05) for Firmicutes, Bacteroidetes, and Cyanobacteria in the NC treatment; Proteobacteria and Actinobacteria in the CO treatment; and Cyanobacteria in the XPC treatment. The most abundant phyla identified in both trials were in accordance with that of previous research (Salanitro et al., 1974, 1978; Wei et al., 2013): Firmicutes (Trial 1: 87.21%; Trial 2: 50.13%), Proteobacteria (Trial 1: 7.71%; Trial 2: 3.43%), and Bacteroidetes (Trial 1: 1.54%; Trial 2: 43.92%). Previous studies using culture-independent methods, targeting the 16S rRNA gene had indicated dominance of the cecal microbiota by Firmicutes (Amit-Romach et al., 2004; Wei et al., 2013).

**FIGURE 3:**
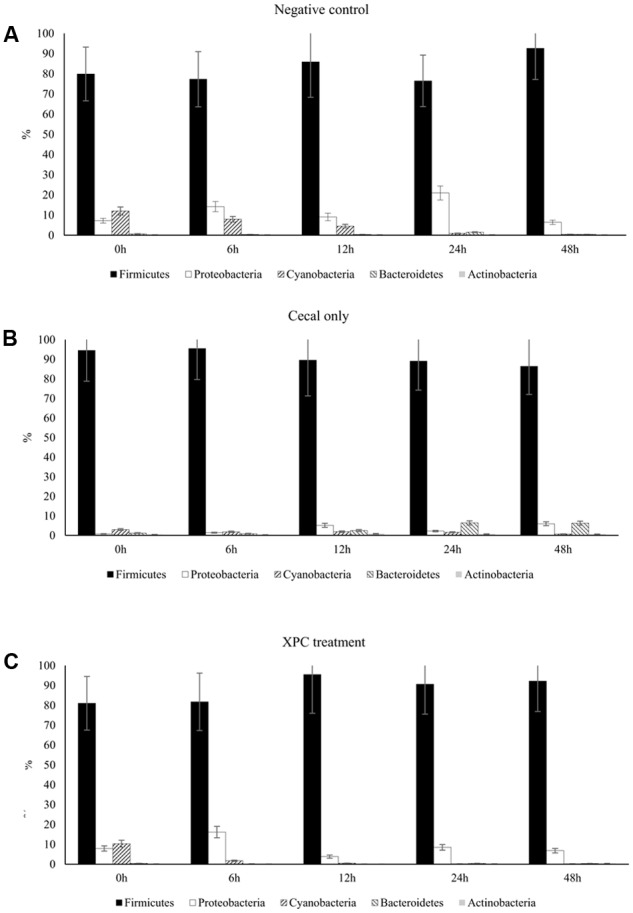
Phylum taxa summary of **(A)** negative control, **(B)** cecal only, and **(C)** XPC treatment in Trial 1. Incubation medium included 0.1 g of cecal contents.

**FIGURE 4:**
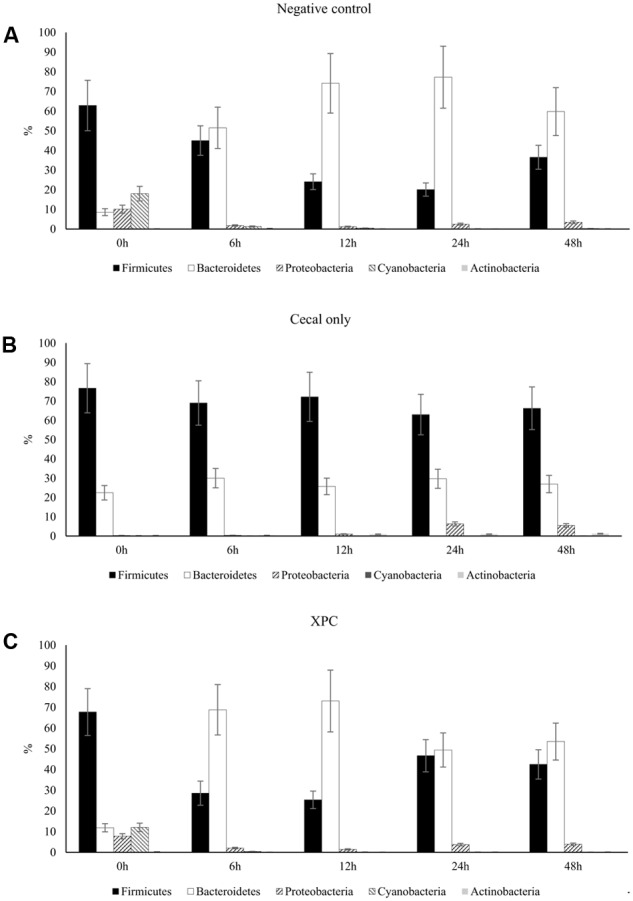
Phylum taxa summary of **(A)** negative control, **(B)** cecal only, and **(C)** XPC treatment in Trial 2. Incubation medium included 0.1 g of cecal contents.

Similar to the Trial 2 results of the current research, Firmicutes and Bacteroidetes have been identified as the most abundant phyla in the chicken cecal microbiota in culture-based studies (Salanitro et al., 1974, 1978). The relative abundances of Proteobacteria and Bacteroidetes both in Trial 2 as well as in previous studies were considered to be more variable (at the expense of the abundance of Firmicutes) with either bacterial community populations ranging from less than 10% to greater than 30% abundance (Zhu et al., 2002; Wei et al., 2013). The largest variation among trials was observed in Bacteroidetes, where the observed abundance in Trial 1 was less than had been previously observed (Wei et al., 2013). The variation observed between the trials suggest that there may have been uncontrolled environmental factors that resulted in distinct phyla abundances among all nine broilers utilized in Trial 1 from the nine broilers used in Trial 2. Trial 2 results are more typical of results observed within the lifespan of the cecal microbiota of healthy chickens (Zhu et al., 2002; Lu et al., 2003). According to the taxa summary in Trial 1, CO group exhibited more Bacteroidetes compared to other groups at 24 and 48 h of incubation time (*P* < 0.05, **Figures 3A–C**). In Trial 2, XPC and NC groups exhibited the highest Firmicutes level at 0 h (*P* < 0.05) but Bacteroidetes overtook Firmicutes after 6 h (**Figures 4A–C**).

**FIGURE 5:**
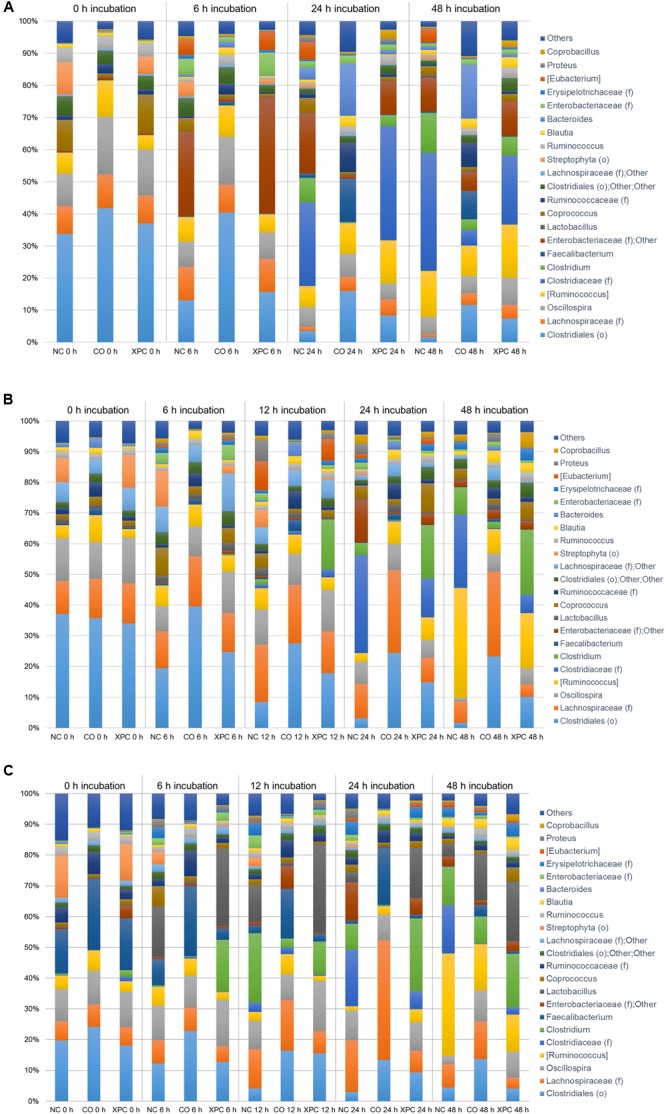
Genus taxa summary of **(A)** 14 days, **(B)** 28 days, and **(C)** 42 days in Trial 1. Incubation medium included 0.1 g of cecal contents.

The relative abundance of major bacteria at the genera level in NC, CO, and XPC treatment containing either 14, 28, or 42 days old chicken ceca in Trial 1 is shown in **Figures 5A–C**, respectively. The top five genera were Clostridiales (order level), *Clostridiaceae* (family level), *Ruminococcus* (taxonomically proposed but not confirmed), *Oscillospira, Enterobacteriaceae* (family level); Others in samples containing 14 days ceca, included *Clostridiales* (order level), *Lachnospiraceae* (family level), *Oscillospira, Ruminococcus* (taxonomically proposed but not confirmed), and *Clostridiaceae* (family level) in samples containing 28 days ceca, and Clostridiales (order level), *Lachnospiraceae* (family level), *Oscillospira, Lactobacillus*, and *Faecalibacterium* in samples containing 42 days ceca. Clostridiales (order level) was the most predominant bacteria and its relative abundance generally decreased along with the incubation time regardless of chicken age or treatments. In contrast, *Clostridiaceae* (family level) populations were only a small percentage of the total microbial communities in the earlier time intervals (0 and 6 h incubation samples: 0.02 to 0.08% in 14 days, 0.01 to 1.19% in 28 days, and 0.13 to 3.14% in 42 days) but they became more predominant in the later samples time intervals (24 and 48 h incubation samples: 0.19 to 36.80% in 14 days, 0.31 to 31.96% in 28 days, and 0.13 to 18.42% in 42 days). There were no significant changes (*P* > 0.05) by treatment in the relative abundance of *Faecalibacterium* and *Lactobacillus* which are generally considered as beneficial bacteria.

**FIGURE 6:**
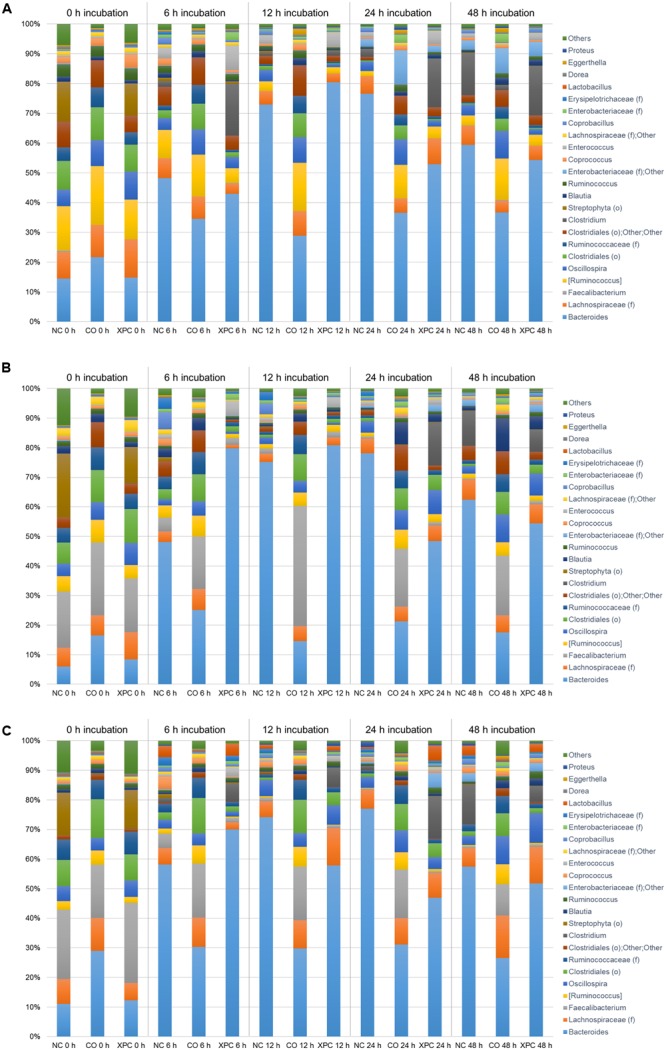
Genus taxa summary of **(A)** 14 days, **(B)** 28 days, and **(C)** 42 days in Trial 2. Incubation medium included 0.1 g of cecal contents.

**Figures 6A–C** represent the relative abundance of major bacteria at the genus level identified in NC, CO, and XPC treatments containing either 14, 28, or 42 days of chicken ceca in Trial 2. Relative abundance of major bacterial groups in Trial 2 at the genus taxonomic level differed with those of Trial 1. The most predominant genus was *Bacteroides* with 45.18, 44.22, and 44.21% for 14, 28, and 42 days samples, respectively and its percentage was increased in conjunction with incubation time. For example, 0 h incubation samples containing 14 days ceca yielded a low abundance of *Bacteroides* with 17.01% but their levels were significantly increased (*P* < 0.05) to 47.38 (6 h), 60.76 (12 h), 55.36 (24 h), and 50.12% (48 h). Similar patterns were also observed in samples containing 28 and 42 days ceca. Other genera such as *Ruminococcus* (taxonomically proposed but not confirmed), *Faecalibacterium, Lachnospiraceae* (family level), Clostridiales (order level), and *Oscillospira* also belonged to the dominant bacterial groups similar to Trial 1. *Faecalibacterium* and *Lactobacillus* exhibited similar levels of relative abundance in the control and XPC treatment groups. *Lactobacillus* is a common genus present in cecal contents of healthy poultry and it is well-known for supporting the ecological balance of cecal microbial populations. Faecalibacterium, butyric acid producing bacterium, is well-known for a beneficial commensal relationship associated with the host gut health. The increases of both bacterial groups in XPC treatment suggests that addition of XPC in the broiler diet could selectively support beneficial cecal microbiota populations. However, this will need to be examined further using *in vivo* studies where XPC is included in the diets fed to broilers throughout the growth cycle at these levels of supplementation.

The temporal effects on species diversity and richness as evaluated by observed OTUs revealed that the measurements followed the trend of directly increasing with age in both trials. Trial 1 revealed less variation while Trial 2 revealed significant increases (*P* < 0.05) at 28 and 42 days compared to 14 days (data not shown). Increasing cecal microbiota complexity that directly relates to sampling age has been observed in previous studies on poultry intestinal microbiota characterization (Salanitro et al., 1974; Danzeisen et al., 2013; Oakley et al., 2014). Meimandipour et al. (2011) provided evidence for this based on the variation in the production of SCFA observed in the ceca across various sampling ages. The continued projection upward rather than a plateau indicates that further subsampling of sequences would increase the species richness and increase the numbers of OTUs. The trend when evaluating the OTU rarefaction curves revealed the CO and NC groups in Trial 1 to consistently possess higher levels compared to NC in Trial 2 (*P* < 0.05).

**FIGURE 7:**
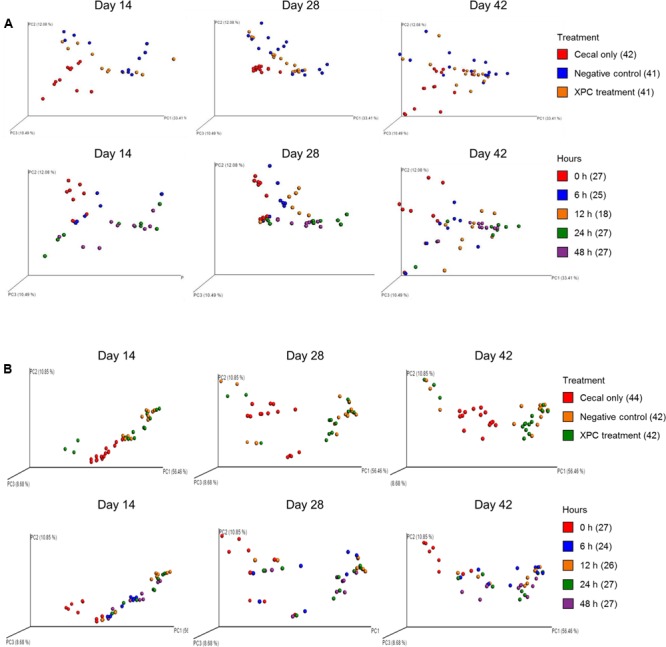
UniFrac weighted PCoA plots of **(A)** Trial 1 and **(B)** Trial 2. For label on treatment, cecal only (red), negative control (blue) and XPC (orange). For label on hour, 0 h (red), 6 h (blue), 12 h (orange), 24 h (green), and 48 h (purple).

The weighted PCoA plots indicated similar clustering of the NC and XPC groups in contrast to the CO group in Trial 1 (**Figure 7A**). In addition, shifting patterns were observed as a function of incubation time. In Trial 2, NC and XPC exhibited similar clustering patterns compared to CO, to those observed in Trial 1. Finally, incubation time of 6, 12, 24, and 48 h exhibited distinct clustering patterns in bacterial populations compared to 0 h sample clustering (**Figure 7B**).

## Conclusion

Research efforts directed toward evaluating the effectiveness of in feed supplements seeking to fill the gap left due to the industry wide trend of AGP removal from animal feed now becoming commonplace. The current study reviewed the influence of XPC in the presence of cecal contents on the inhibition of *S.* Typhimurium and reported that there was initial prevention, however, the level of reduction eventually became equal among all of the treatments containing cecal contents. These findings suggest the ability of XPC to accelerate the rate at which *S.* Typhimurium and possibly other pathogens are inhibited by the cecal microbiota. Because AGPs have thus far indicated the ability to promote bird growth while limiting pathogens in poultry, it would be beneficial to characterize the intestinal microbiome when bird diets are supplemented with AGPs, and compare the resulting intestinal microbiome response to that of birds fed various feed supplements (probiotics, prebiotics, synbiotics). For the poultry industry to utilize the current research, it may be beneficial to accelerate development of the intestinal microbial complexity of the broiler host by using supplements that interact both directly and indirectly with the host intestinal microbiome and promote a diversity that is more related to the microbiome characteristic of mature birds.

## Author Contributions

SP, PMR, SMR, and SCR designed the experiment, SP, SK, SL, SMR, PMR performed the experiment, SL, SP, SMR, PMR, and SK analyzed date, SL, SP, SK, SMR, PMR, and SCR wrote the manuscript. HP, DM, SK, PMR, and SCR reviewed the manuscript.

## Conflict of Interest Statement

The authors declare that the research was conducted in the absence of any commercial or financial relationships that could be construed as a potential conflict of interest.
